# Supplementary material for: Hardware-tailored logical Clifford circuits for stabilizer codes
Source: npj Quantum Inf. 2026 Nov 12;12(1):185. doi: 10.1038/s41534-026-01404-x (PMC13759319; doi:10.1038/s41534-026-01404-x)
Supplement: Supplementary file 1 [file 41534_2026_1404_MOESM1_ESM.pdf]

Supplementary Material (SM) to
Hardware-tailored logical Clifford circuits for stabilizer codes

Eric J. Kuehnke,[1,*] Kyano Levi,[1] Joschka Roffe,[1,2] Jens Eisert,[1] and Daniel Miller[1,3]

[1] *Dahlem Center for Complex Quantum Systems, Freie Universität Berlin, 14195 Berlin, Germany*
[2] *School of Informatics, The University of Edinburgh, EH8 9AB Edinburgh, Scotland, UK*
[3] *Institute for Theoretical Nanoelectronics (PGI-2), Forschungszentrum Jülich, 52428 Jülich, Germany*

(Dated: October 27, 2026)

I. EMPIRICAL RUNTIMES OF THE IQCP SOLVER

In this section, we present runtimes of our open-source implementation of the Gurobi-based IQCP solver for Eq. (13) of the main text. Note that this runtime should be interpreted as the classical preprocessing cost associated with constructing a hardware-tailored circuit implementation of a desired logical Clifford gate for a given stabilizer code. Making such circuits weakly fault-tolerant in a subsequent step requires applying the techniques outlined in methods section "Weak fault tolerance with flag gadgets". The IQCP solver runtimes for constructing all 720 Clifford gates for the $[\![4, 2, 2]\!]$ iceberg code are reported in section "First use case: $[\![4, 2, 2]\!]$ iceberg code" of the main text. The runtimes for constructing the remaining circuits from the main text, along with two additional examples reported only here, are shown in Table S1. The IQCP solver by Gurobi proceeds in two steps. First, it constructs *some* feasible point (in the mixed integer/binary parameter space of B_i, G_i variables and slack variables; see Eq. (13) in the main text) for the IQCP, then it proves optimality (and potentially replaces the initial feasible point by a better one). The times required for this are shown in Table S1 in the columns "Found" and "Optimality", respectively, where the latter refers to the total runtime (including the time for constructing the initial feasible point). For some target circuits, we solve the IQCP for more than one length l of the ansatz circuit U_{A_l} in Eq. (2) of the main text, which results in a solution with a different CZ count and a different runtime.

Code	Gate	Length l	CZ count	Found	Optimality
$[\![12, 2, 3]\!]$	$\overline{CX_{2,1}}$	2	11	45 min	119 min
		3	9	76 h	/
$[\![8, 3, 2]\!]$	$\overline{H_1}$	3	7	8 min	51 min
		4	7	21 h	/
	$\overline{H_{1,2}^{\otimes 2}}$	3	8	86 min	90 min
		4	8	90 h	163 h
	$\overline{H_{1,2,3}^{\otimes 3}}$	3	15	20 h	/
	$\overline{S_1}$	1	1	< 1 s	< 1 s
		3	1	40 s	40 s
	$\overline{(HS)}_1$	3	6	61 min	66 min
$[\![16, 6, 2]\!]$	$\overline{CZ_{1,4}}$	3	5	48 min	119 min

TABLE S1: Empirical runtimes of the Gurobi-based IQCP solver used to construct hardware-tailored logical gate implementations for various codes presented in the main text. All calculations were carried out on four cores of an Intel Xeon CPU E5-2695 v2 @2.40 GHz with 20 GB of RAM allocated. Note that different logical gates can be constructed in parallel, hence, circuits for a meaningful experiment can often be obtained within hours or days.

The second row in Table S1 shows that the construction of the circuit in Fig. 2 of the main text—which implements a controlled-X gate from qubit 2 to qubit 1 for the $[\![12, 2, 3]\!]$ twisted toric code—required approximately three days. By reducing the ansatz length to $l = 2$, a similar circuit implementation with two additional CZ gates can be constructed in only 45 minutes, and the optimality (in terms of CZ count and for the given ansatz) is proven in an additional 74 minutes, as shown in the first row of Table S1.

Next, consider the $[\![8, 3, 2]\!]$ code as defined in the main text. The circuits presented in Fig. 3 of the main text were constructed for an ansatz length of $l = 3$, with solver runtimes ranging from 40 seconds for the logical S gate to 20 hours for the circuit implementing a Hadamard gate on all three logical qubits. We observe that, for a fixed ansatz length, the solver often runs significantly faster when a circuit implementation with a low CZ count exists. In the extreme case, the logical S gate requires only a single CZ gate, and its circuit can be constructed with $l = 1$ in under a second. Note that this simple circuit implementation can also be obtained using the methods of Ref. [1], since the S gate is diagonal in the computational basis. For the more challenging Hadamard circuits, we also apply our solver with an increased ansatz length of $l = 4$. However, despite the significantly longer runtime, we do not obtain circuits with a lower CZ count.

Besides the circuits from Fig. 3 of the main text, we also construct a logical $\overline{HS}$ gate (not shown) for the $[\![8, 3, 2]\!]$ code, which requires approximately 1 hour of preprocessing time and uses six CZ gates with an ansatz length of $l = 3$. Compared to the sequence in which the S gate and then the Hadamard gate are applied, the direct implementation of the combined logical $\overline{HS}$ gate saves two CZ gates. This demonstrates the flexibility of our framework in constructing fully compiled circuits, enabling, for instance, faster access to the Y basis—an improvement with important applications [2].

* eric.kuehnke@fu-berlin.de

In the last row of Table S1, we report a runtime of 48 minutes for constructing a logical CZ gate between two distinct blocks of the $[\![8, 3, 2]\!]$ code. This circuit implementation, available on GitHub [3], provides another example of the flexibility of our framework in directly tackling the addressability problem of delocalized logical qubits. Since the two code blocks comprise 16 physical qubits in total, this substantiates the claim made in the abstract about the scalability of our methods.

To complement the code-specific runtimes in Table S1, we study the dependence of the computational resource costs on the code size using random stabilizer codes with fixed encoding rate $k/n = 1/2$. For each $n \in \{4, 6, 8, 10, 12, 14\}$, we sample 100 codes with parameters $[\![n, n/2, d]\!]$ and $d \geq 2$, by rejecting and resampling instances with $d = 1$. For each code, we compile the logical Hadamard gate, $\overline{H}_1$, which acts on the first logical qubit. This is achieved by solving Eq. (13) of the main text, where we use an ansatz length of $l = 1$ and unrestricted connectivity as hyperparameters. In this way, we assess the dependence of the computational resource costs as a function of the code size.

Figure S1 shows the empirical distributions of solver runtimes (bottom plot) and recorded peak RAM usage (top plot; left y-axis), together with the numbers of IQCP variables and constraints (top plot; right y-axis). We configured Gurobi with `NodefileStart=0.5`, enabling compressed storage of branch-and-bound tree nodes on disk once their memory consumption exceeds 0.5 GB. Memory usage (excluding disk space) was obtained from Slurm's `MaxRSS` (maximum resident set size) records with a 30-second accounting interval. Since memory requirements are unavailable if an IQCP is solved too quickly, the memory distributions describe only the subset of instances—the fraction for which this information is available is reported in the figure (orange annotations over the violins).

We see in Figure S1 that runtimes and recorded memory usage both increase with n, with substantial variation between instances. The runtime distributions also illustrate the additional computational effort required to certify CZ count optimality within the chosen ansatz. A feasible circuit was found for every sampled instance, while optimality was proven for all instances with $n \leq 12$ and for 95 of the 100 instances with $n = 14$ within the configured Gurobi time limit of $1201400\,\mathrm{s} \approx 13.9\,\mathrm{d}$.

Figure S1 demonstrates the applicability of our method across randomly sampled codes and provides a reference for its computational resource requirements in the benchmarked setting. The large variation between instances also shows that code size alone is insufficient to predict the runtime for a particular compilation problem. Nevertheless, a log-linear fit to the observed minimum total solver runtimes for $n \in \{4, \ldots, 14\}$ extrapolates to approximately 1.4×10^4 s at $n = 30$, suggesting that even at this size favorable instances may require only several hours of computation. Since circuit compilation is a one-time preprocessing step, such runtimes remain practical for repeated experimental use.

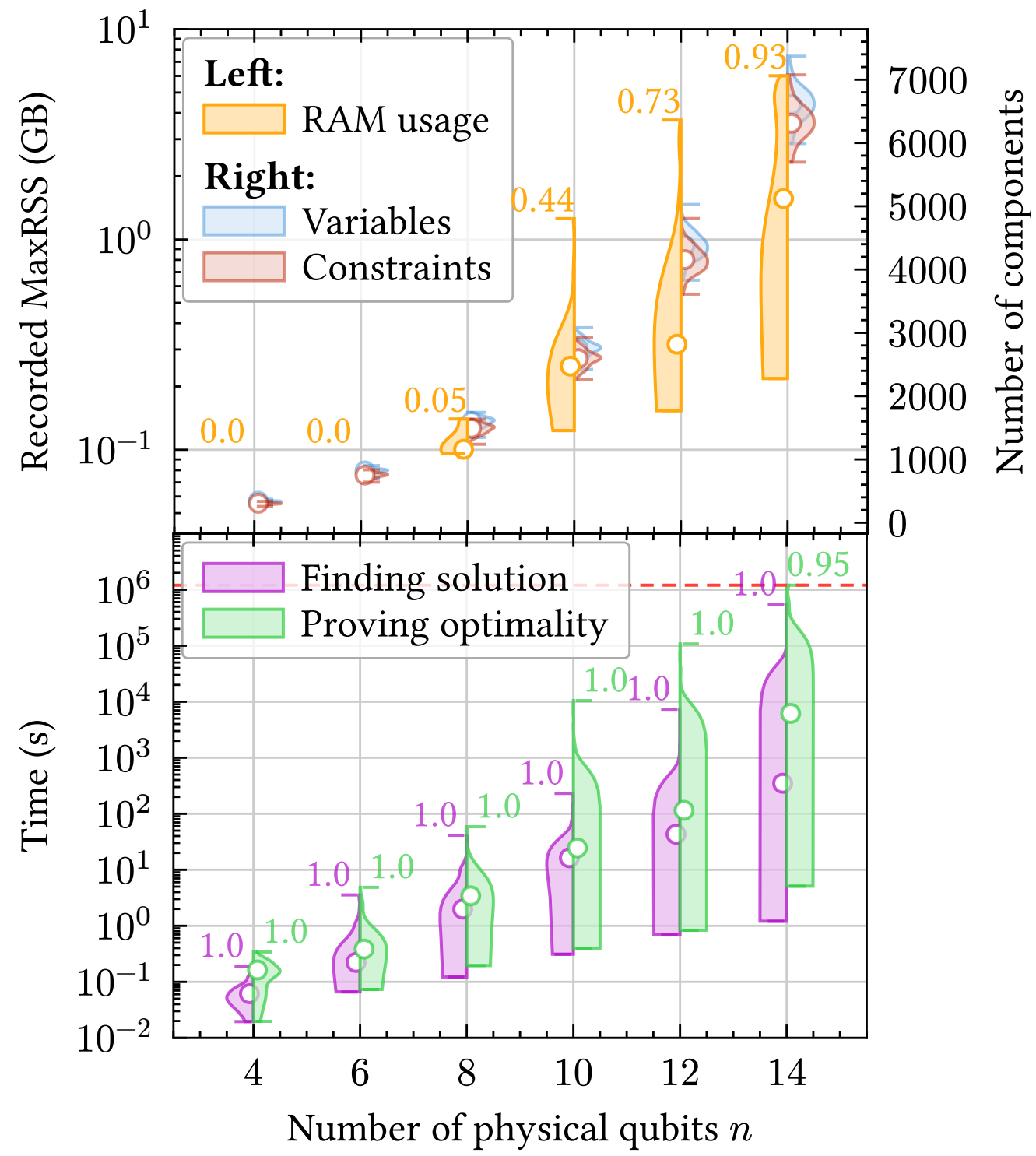

FIG. S1: Empirical computational resource requirements for compiling the logical Hadamard gate $\overline{H}_1$ on 100 random $[\![n, n/2, d]\!]$ stabilizer codes with $d \geq 2$ as a function of n. Violin shapes show the distributions across instances, horizontal bars indicate the maximum and minimum values, and dots mark the median. Upper panel: recorded RAM usage (orange, left y-axis) and numbers of IQCP variables and constraints (blue and red, right y-axis). Memory values are taken from Slurm's `MaxRSS`; orange annotations indicate the fraction of jobs for which memory usage was available. Lower panel: times at which the final reported solutions were found (violet) and total solver runtimes (green), shown on a logarithmic scale (left y-axis). For runs in which optimality was not proven, the corresponding stopping times are shown. Violet and green annotations indicate the fractions of instances for which a feasible circuit was found and CZ count optimality within the chosen ansatz was proven, respectively. The dashed red line indicates the configured Gurobi time limit of approximately 14 days. All calculations were carried out on four cores of an Intel Xeon CPU E5-2695 v2 @2.40 GHz with 20 GB of RAM allocated.

II. PROOF OF THEOREM 1

In this appendix, we prove Theorem 1 from the main text. More precisely, we show that

$$\mathcal{F} = \{F \in \mathrm{Sp}(\mathbb{F}_2^{2n}) \mid F \text{ obeys main text Eq. (6)}\} \quad \text{(S1)}$$

serves as the gauge group for logical Clifford operations whose action is specified only on the logical subspace and may differ outside the code space. Moreover, we prove that

the cardinality of $\mathcal{F}$ is given by the expression in Eq. (7) in the main text.

Proof. Our first claim is that $\mathcal{F}$ is a group. To show this, we write the elements $F \in \mathcal{F}$ in block form as in

$$F = \begin{bmatrix} F^{xx} & 0_n \\ F^{zx} & F^{zz} \end{bmatrix}, \tag{S2}$$

where $0_n \in \mathbb{F}_2^{n\times n}$ denotes the zero matrix. The constraints from Eq. (6) in the main text on the blocks are given by

$$F^{xx} = \begin{bmatrix} \mathbb{1}_k & * & \cdots & * \\ 0 & * & \cdots & * \\ \vdots & \vdots & \ddots & \vdots \\ 0 & * & \cdots & * \end{bmatrix}, \quad F^{zz} = \begin{bmatrix} \mathbb{1}_k & 0 & \cdots & 0 \\ * & * & \cdots & * \\ \vdots & \vdots & \ddots & \vdots \\ * & * & \cdots & * \end{bmatrix}, \tag{S3}$$

$$\text{and} \quad F^{zx} = \begin{bmatrix} 0_k & * & \cdots & * \\ * & * & \cdots & * \\ \vdots & \vdots & \ddots & \vdots \\ * & * & \cdots & * \end{bmatrix}. \tag{S4}$$

Clearly, $F = \mathbb{1}_{2n}$ fulfills these constraints, proving $\mathbb{1}_{2n} \in \mathcal{F}$. Taking the product of two matrices $F, \tilde{F} \in \mathcal{F}$ results in

$$F\tilde{F} = \begin{bmatrix} F^{xx}\tilde{F}^{xx} & 0_n \\ F^{zx}\tilde{F}^{xx} + F^{zz}\tilde{F}^{zx} & F^{zz}\tilde{F}^{zz} \end{bmatrix}. \tag{S5}$$

It is straightforward to verify that $F^{xx}\tilde{F}^{xx}$ and $F^{zz}\tilde{F}^{zz}$ inherit the constraints of Eq. (S3). Similarly, both $F^{zx}\tilde{F}^{xx}$ and $F^{zz}\tilde{F}^{zx}$ obey the constraints of Eq. (S4), which proves $F\tilde{F} \in \mathcal{F}$. This shows that $\mathcal{F}$ is closed under taking products. Hence, it is also closed under taking inverses because $\mathcal{F} \subset \mathbb{F}_2^{2n\times 2n}$ is clearly finite. This proves that $\mathcal{F}$ is a group.

As mentioned in the main text, $\mathcal{F}$ can be understood as the subgroup of non-Pauli Clifford stabilizers of the code space $\mathcal{L}$ of an $[\![n,k,d]\!]$ code. Next we will show that $\mathcal{F}$ is in bijection with the different choices of physical Clifford operators (modulo Paulis and phases) that implement a given logical Clifford gate. In that sense, the elements of $\mathcal{F}$ correspond to different gauges of logical Clifford operators. To distinguish it from the concept of *gauge groups* in subsystem QECCs [4–6], we refer to $\mathcal{F}$ as the *freedom gauge group*.

We need to show that $U_{EC'FE^{-1}}$ implements $U_C \in \mathcal{C}^k$ on the logical level whenever $F \in \mathcal{F}$ and, conversely, that every n-qubit Clifford operation that does so is of the form $U_{EC'FE^{-1}}$ for some $F \in \mathcal{F}$. For both statements we will make use of the fact that the encoding circuit U_E maps Pauli-Z operators on qubits $k+1$ to n to stabilizers $S \in \langle S_1, \ldots, S_{n-k}\rangle$ and arbitrary Pauli operators on qubits 1 to k to logical Pauli operators $\bar{P} \in \langle \bar{X}_1, \bar{Z}_1, \ldots, \bar{X}_k, \bar{Z}_k\rangle$. To make this more precise, we introduce binary vectors $\mathbf{s}_j, \mathbf{s}'_j, \mathbf{x}_i, \mathbf{x}'_i, \mathbf{z}_i, \mathbf{z}'_i \in \mathbb{F}_2^n$ such that $S_{j-k} \propto X^{\mathbf{s}_j} Z^{\mathbf{s}'_j}$, for all $j \in \{k+1, \ldots, n\}$ and $\bar{X}_i \propto X^{\mathbf{x}_i} Z^{\mathbf{x}'_i}$, $\bar{Z}_i \propto X^{\mathbf{z}_i} Z^{\mathbf{z}'_i}$ for all $i \in \{1, \ldots, k\}$, see Eq. (20) in the main text. Then, we have

$$E \begin{bmatrix} \mathbf{e}_i \\ 0 \end{bmatrix} = \begin{bmatrix} \mathbf{x}_i \\ \mathbf{x}'_i \end{bmatrix}, \quad E \begin{bmatrix} 0 \\ \mathbf{e}_i \end{bmatrix} = \begin{bmatrix} \mathbf{z}_i \\ \mathbf{z}'_i \end{bmatrix}, \tag{S6}$$

$$\text{and} \quad E \begin{bmatrix} 0 \\ \mathbf{e}_j \end{bmatrix} = \begin{bmatrix} \mathbf{s}_j \\ \mathbf{s}'_j \end{bmatrix}, \tag{S7}$$

where $\mathbf{e}_l = (\delta_{l,l'})_{l'=1}^n \in \mathbb{F}_2^n$ denotes a standard basis vector.

First, assume we have a Clifford operation $U_M \in \mathcal{C}^n$ that is represented by $M = EC'FE^{-1}$ for some $F \in \mathcal{F}$. We have to show that U_M acts as C on the logical level. For every $j \in \{k+1, \ldots, n\}$, we find that $U_M S_{j-k} U_M^\dagger$ is represented by

$$M \begin{bmatrix} \mathbf{s}_j \\ \mathbf{s}'_j \end{bmatrix} = E \sum_{l=k+1}^{n} F_{l+n,j+n} \begin{bmatrix} 0 \\ \mathbf{e}_l \end{bmatrix}, \tag{S8}$$

where we have used Eq. (S7) and the fact that $F^{xz} = 0$, which holds by definition of $F \in \mathcal{F}$. Again applying Eq. (S7), we further find

$$M \begin{bmatrix} \mathbf{s}_j \\ \mathbf{s}'_j \end{bmatrix} = \sum_{l=k+1}^{n} F_{l+n,j+n} \begin{bmatrix} \mathbf{s}_l \\ \mathbf{s}'_l, \end{bmatrix} \tag{S9}$$

which, after a suitable Pauli-frame correction to U_M, implies $U_M S_{j-k} U_M^\dagger \in \mathcal{S}$. Therefore, Lemma 3 from methods section "Characterization of logical Clifford gates" applies, and shows that U_M is a logical operator. To determine its logical action, let us first compute the vectors that represent $U_M \overline{X_i} U_M^\dagger$ for all $i \in \{1, \ldots, k\}$, that is

$$M \begin{bmatrix} \mathbf{x}_i \\ \mathbf{x}'_i \end{bmatrix} = EC' \begin{bmatrix} \mathbf{e}_i \\ 0 \end{bmatrix} + \sum_{l=k+1}^{n} F_{l+n,i} \begin{bmatrix} \mathbf{s}_l \\ \mathbf{s}'_l \end{bmatrix}. \tag{S10}$$

The first term in Eq. (S10) represents the logical Pauli operator into which the i-th Pauli-X operator is transformed, while the second term reflects that this mapping is defined modulo stabilizers. Similarly, we find

$$U_M \overline{Z_i} U_M^\dagger = \overline{U_C Z_i U_C^\dagger} S'_i \tag{S11}$$

for some $S'_i \in \mathcal{S}$. Therefore, Lemma 4 from methods section "Characterization of logical Clifford gates" applies, and we have shown that U_M acts as $U_C \in \mathcal{C}^k$ on the logical level.

Next, we show the converse that every Clifford operation $U_M \in \mathcal{C}^n$ that implements $U_C \in \mathcal{C}^k$ on the logical level can be written as $M = EC'FE^{-1}$ for some freedom matrix $F \in \mathcal{F}$. Our strategy is to translate the constraints on M imposed by Lemmata 3 and 4 from methods section "Characterization of logical Clifford gates" into the constraints on F that are shown in Eq. (6) in the main text. To this end,

we define the freedom matrix $F = C'^{-1}E^{-1}ME$. To prove $F \in \mathcal{F}$, we first apply F to the unit vector corresponding to the unencoded i-th logical Pauli-X operator from Eq. (S6). By Eq. (S6), this yields

$$F \begin{bmatrix} \mathbf{e}_i \\ 0 \end{bmatrix} = C'^{-1}E^{-1}M \begin{bmatrix} \mathbf{x}_i \\ \mathbf{x}'_i \end{bmatrix} \tag{S12}$$

Next, we apply Lemma 4 from methods section "Characterization of logical Clifford gates" to arrive at

$$F \begin{bmatrix} \mathbf{e}_i \\ 0 \end{bmatrix} = \begin{bmatrix} \mathbf{e}_i \\ 0 \end{bmatrix} + \sum_{l=k+1}^{n} F_{l+n,i} \begin{bmatrix} 0 \\ \mathbf{e}_l \end{bmatrix}, \tag{S13}$$

which proves the restrictions on F shown in Eq. (6) in the main text for columns 1 to k. Similarly, by repeating the calculation for the logical Pauli-Z operators, we obtain the corresponding restrictions for columns $n+1$ to $n+k$. Finally, we analyze the constraints on F that are imposed by how U_M is allowed to transform stabilizer operators. By Lemma 3 from methods section "Characterization of logical Clifford gates", we have

$$\begin{aligned} F \begin{bmatrix} 0 \\ \mathbf{e}_j \end{bmatrix} &= C'^{-1}E^{-1}M \begin{bmatrix} \mathbf{s}_j \\ \mathbf{s}'_j \end{bmatrix} \\ &= \sum_{l=k+1}^{n} F_{l+n,j+n} \begin{bmatrix} 0 \\ \mathbf{e}_l \end{bmatrix}, \end{aligned} \tag{S14}$$

which proves that also columns $n+k+1$ to $2n$ have to be of the form given in Eq. (6) in the main text. Since there are no constraints, besides $F \in \mathrm{Sp}(\mathbb{F}_2^{2n})$, on columns $k+1$ to n, this finishes the proof of $F \in \mathcal{F}$.

Finally, let us compute the order $|\mathcal{F}|$ of the freedom gauge group. Because every freedom matrix $F \in \mathcal{F}$ is invertible, the same must be true about the block matrix F^{zz}. By Eq. (S3), this is the case iff the submatrix of size $(n-k) \times (n-k)$ in the bottom right of F^{zz} is invertible. Besides this, there are no further constraints on the column vectors of F^{zz}. Thus, there are

$$|\mathbb{F}_2^{n-k}|^k \times |\,\mathrm{GL}(\mathbb{F}_2^{n-k})| = 2^{k(n-k)} \prod_{i=0}^{n-k-1} (2^{n-k} - 2^i) \tag{S15}$$

possible choices for F^{zz}. Next, we write out the condition $F \in \mathrm{Sp}(\mathbb{F}_2^{2n})$, i.e., $F^T \left[\begin{smallmatrix} 0 & \mathbb{1} \\ \mathbb{1} & 0 \end{smallmatrix}\right] F = \left[\begin{smallmatrix} 0 & \mathbb{1} \\ \mathbb{1} & 0 \end{smallmatrix}\right]$, which yields

$$(F^{xx})^T F^{zz} = \mathbb{1} \quad \text{and} \tag{S16}$$

$$\left((F^{xx})^T F^{zx}\right)^T = (F^{xx})^T F^{zx}. \tag{S17}$$

By Eq. (S16), the submatrix $F^{xx} = ((F^{zz})^T)^{-1}$ is uniquely determined through the choice of F^{zz}. Equation (S17) means that $(F^{xx})^T F^{zx}$ must be a symmetric matrix. Since we can regard $(F^{xx})^T$ as a bijective map, the number of allowed choices for $(F^{xx})^T F^{zx}$ and F^{zx} are identical. There are in total $2^{n(n+1)/2}$ symmetric binary $n \times n$-matrices. However, not all of them are allowed by Eq. (6) in the main text. Rows 1 to k of F^{zz} map columns 1 to k of $(F^{xx})^T F^{zx}$ to zero in F^{zx}, since $F^{zz}[(F^{xx})^T F^{zx}] = F^{zx}$. This reduces the number of free variables of $(F^{xx})^T F^{zx}$ by $k(k+1)/2$ for a given choice of F^{zz} and F^{xx}. In total, this shows that the order of the freedom gauge group $\mathcal{F}$ is indeed given by the expression in Eq. (7) in the main text, which finishes the proof of Theorem 1. □

This proof contains an explicit (albeit somewhat opaque) enumeration of all elements in the freedom gauge group $\mathcal{F}$. To better understand the role of $\mathcal{F}$, we rearrange Eq. (7) in the main text into

$$|\mathcal{F}| = \underbrace{2^{2k(n-k)}}_{(1)} \underbrace{2^{(n-k)(n-k+1)/2}}_{(2)} \underbrace{\prod_{i=0}^{n-k-1} \left(2^{n-k} - 2^i\right)}_{(3)}. \tag{S18}$$

Factor (3) in Eq. (S18) is the number of ways in which the $n-k$ stabilizer generators can be mapped to a different choice of stabilizer generators. Similarly, factor (1) is the number of ways to correctly transform $2k$ logical Pauli operators modulo stabilizers, while factor (2) characterizes the additional freedom provided by the transformation of Pauli errors.

III. CIRCUIT-LEVEL NOISE SIMULATION

In this appendix, we provide details about the circuit-level noise simulations that we carried out to produce Fig. 4 in the main text.

The goal of these simulations is twofold: to provide a numerical sanity check that our logical Hadamard circuits for the $[\![8,3,2]\!]$ code are indeed weakly fault-tolerant (FT), and to compare their performance against an existing protocol [7]. All simulations were carried out with Stim [8], using a circuit-level noise model in which all error probabilities (of gates, measurements, resets, etc.) are proportional to a single physical error parameter called p. This parameter serves as the x-axis of Fig. 4 in the main text. Our noise model is inspired by the one in Tab. 3 of Ref. [2]. However, we find it convenient to slightly modify it as follows. Besides controlled-Z gates, we also include controlled-X and -Y gates in the set of available gates. For these gates, we assume the same two-qubit depolarizing noise as for the controlled-Z gate. Besides measurements and resets in the Z-basis, we also include the same operations in the X-basis. Here, we replace bit errors with phase errors, while keeping the same error rates. Note that the conclusions drawn in the main text are not restricted to this specific choice of noise model.

Before running a circuit-level error simulation, we have to specify a circuit that includes (1) weakly FT state preparation, (2) weakly FT logical gates, (3) weakly FT stabilizer measurements, and (4) weakly FT measurement of logical Paulis.

Let us explain how we select these components to study logical Hadamard gates for the $[\![8,3,2]\!]$ code. First, we need

weakly FT state preparation circuits. To prepare the logical state vector $|\overline{0,0,0}\rangle$, we can initialize every physical qubit in $|0\rangle$ and perform a weakly FT measurement (see below) of the only X-type stabilizer operator, $X^{\otimes 8}$. If instead we wish to prepare $|\overline{+,+,+}\rangle$, we use the circuit shown in Fig. 3 of Ref. [9]. These circuits are also available on GitHub [10].

Second, we need FT logical gates. For this, we work either with our new weakly FT gates constructed in the main text or with the teleportation-based constructions from Ref. [7], which are also available on GitHub [11]. Recall from Definition 1 in methods section "Weak fault tolerance with flag gadgets" that we define a logical gate for a code with distance $d = 2$ to be weakly FT if every single error is flagged, its residual error is detectable, or it has a trivial logical action. A detectable error, however, is not the same as a detected error. To effectively remove error mechanisms from a circuit, we need to detect the errors by performing a round of stabilizer measurements. For the $[\![8,3,2]\!]$ code, this can be ensured by employing a flag construction similar to that in Lemma 5 in methods section "Weak fault tolerance with flag gadgets", with the roles of flags 1 and 2 taken by the auxiliary qubit and the flag, respectively. Finally, we can implement the weakly FT measurement of logical Pauli operators by performing a round of stabilizer measurements, followed by reading out physical qubits in the basis corresponding to any X- or Z-type representative of the logical operator. Here, we save resources by inferring the syndromes of stabilizers whose eigenvalues can be reconstructed as parities of the physical measurement outcomes, rather than measuring those stabilizers with a weakly FT stabilizer extraction circuit, using flags. For example, to perform a weakly FT measurement of $\overline{Z_1}$, $\overline{Z_2}$, and $\overline{Z_3}$, we execute a flag-based weakly FT stabilizer measurement for $X^{\otimes 8}$ before we read out all eight physical qubits in the computational basis. For the weakly FT measurement of $\overline{X_1}$, $\overline{X_2}$, and $\overline{X_3}$, we could, in principle, proceed similarly. However, instead we execute the time-reversed circuit of the weakly FT state preparation circuit for $|\overline{+,+,+}\rangle$ from Ref. [9]. Here, weak fault tolerance follows from the fact that every fault occurring during the reversed circuit propagates to at most a detectable error. These circuits are also available on GitHub [12].

Finally, we combine these building blocks into weakly FT circuits that map $|\overline{0,0,0}\rangle$ to $|\overline{+,+,+}\rangle$ and vice versa. All gates, measurements (including those of the flag qubits in the stabilizer measurements), and resets are subject to the noise model; no element is treated as noise-free. The circuits used in the simulations are available online on GitHub [3].

[1] M. A. Webster, A. O. Quintavalle, and S. D. Bartlett, Transversal diagonal logical operators for stabiliser codes, New J. Phys. **25**, 103018 (2023).

[2] C. Gidney, Inplace access to the surface code Y basis, Quantum **8**, 1310 (2024).

[3] E. J. Kuehnke *et al.*, GitHub: hardware-tailored logical gates, https://github.com/erkue/htlogicalgates (2026).

[4] D. A. Lidar and T. A. Brun, *Quantum error correction* (Cambridge University Press, 2013).

[5] C. Vuillot, L. Lao, B. Criger, C. G. Almudéver, K. Bertels, and B. M. Terhal, Code deformation and lattice surgery are gauge fixing, New J. Phys. **21**, 033028 (2019).

[6] M. Li, D. Miller, M. Newman, Y. Wu, and K. R. Brown, 2D compass codes, Phys. Rev. X **9**, 021041 (2019).

[7] Y. Wang *et al.*, Fault-tolerant one-bit addition with the smallest interesting color code, Sci. Adv. **10**, eado9024 (2024).

[8] C. Gidney, Stim: a fast stabilizer circuit simulator, Quantum **5**, 497 (2021).

[9] D. Honciuc Menendez, A. Ray, and M. Vasmer, Implementing fault-tolerant non-Clifford gates using the [[8,3,2]] color code, Phys. Rev. A **109**, 062438 (2024).

[10] See color832_prepare_000.txt and color832_prepare_ppp.txt in https://github.com/erkue/htlogicalgates/tree/main/examples.

[11] See files color832_wang_H0.txt and color832_wang_H01.txt in https://github.com/erkue/htlogicalgates/tree/main/examples.

[12] See files color832_read_000.txt and color832_read_ppp.txt in https://github.com/erkue/htlogicalgates/tree/main/examples.
